# Regeneration of amputated mice digit tips by including Wnt signaling pathway with CHIR99021 and IWP-2 chemicals in limb organ culture system

**DOI:** 10.22038/ijbms.2024.76957.16643

**Published:** 2024

**Authors:** Leila Taghiyar, Fatemeh Bijarchan, Mahshad Doraj, Mohamadreza Baghban Eslaminejad

**Affiliations:** 1 Department of Stem Cells and Developmental Biology, Cell Science Research Center, Royan Institute for Stem Cell Biology and Technology, ACECR, Tehran, 1665659911, Iran; 2 Department of Developmental Biology, University of Science and Culture, Tehran, Iran

**Keywords:** Bone formation, Organ culture, Proliferation, Regeneration, Wnt signaling pathway

## Abstract

**Objective(s):**

Mammals have limited limb regeneration compared to amphibians. The role of Wnt signaling pathways in limb regeneration has rarely been studied. So, this study aimed to investigate the effect of Wnt-signaling using chemicals CHIR99021 and IWP-2 on amputated mice digit tips regeneration in an *in vitro* organ culture system.

**Materials and Methods:**

The distal phalanx of paws from C57BL/6J mouse fetuses at E14.5, E16.5, and E18.5 was amputated. Then, the hands were cultured for 7 days. Subsequently, paws were treated with 1–50 µg/ml concentration of CHIR99021 and 5–10 µg/ml concentration of IWP-2. Finally, the new tissue regrowth was assessed by histological analysis, immunohistochemistry for BC, TCF1, CAN, K14, and P63 genes, and beta-catenin and Tcf1 genes were evaluated with RT-qPCR.

**Results:**

The paws of E14.5 and E16.5 days were shrinkaged and compressed after 7 days, so the paws of 18.5E that were alive were selected. As a result, newly-grown masses at digit tips were observed in 25 and 30 µl/ml concentrations of the CHR99021 group but not in the IWP2 treatment (*P<0.05; **P<0.01). qRT-PCR analysis confirmed the significant up-regulation of beta-catenin and Tcf1 genes in CHIR99021 group in comparison to the IWP-2 group (*P*<0.05). Moreover, Alcian-blue staining demonstrated the presence of cartilage-like tissue at regenerated mass in the CHIR group. In immunohistochemistry analysis beta-catenin, ACN, Keratin-14, and P63 protein expression were observed in digit tips in the CHIR-treated group.

**Conclusion:**

By activating the Wnt signaling pathway, cartilage-like tissue formed in the blastema-like mass in the mouse’s amputated digit tips.

## Introduction

Unlike amphibians, limb regeneration is not complete in mammalians such as mice and humans and just occurs immediately after birth into digit tips (1). On the other hand, limb regeneration consists of several related events such as the activation of pro-inflammatory response, and wound healing that is followed by scar formation in response to amputation of the appendages in adulthood (2, 3). Accordingly, each step is controlled by numerous signaling pathways that are crucial signaling pathways during limb development (4-6). The major signaling pathways such as the Wingless/integrated signaling pathway (WNT), fibroblast growth factor (FGF), and bone morphogenic protein (BMP) signaling activate in the apical ectoderm ridge (AER) at the dorsoventral (DV) and zone of polarizing activity (ZPA) that is located in the posterior mesenchyme of the limb bud (7, 8). Subsequently, the interaction of Ecto-mesodermal layers Also, stimulation and maintenance of FGF, BMP, and WNT expression together causes formation and elongation of limbs (9). 

In embryogenesis, Wnt signaling pathways have a serious role in several developmental processes and are important in cell turnover and adult tissue homeostasis (10). Also, Wnt signaling has also a key action in cell proliferation, differentiation, and cell survival in important developmental organogenesis such as limbs, bone, and cartilage tissues (11). The dysregulation of Wnt signaling is accurately correlated to some diseases including developmental disability and cancer (12, 13). Three different Wnt signaling pathways have been described so far; one canonical pathway is also known as the Wnt/β-catenin pathway, the Wnt/Ca2+-dependent pathway and the Wnt/planar cell polarity (PCP) pathway as two non-canonical pathways (14). 

Accordingly, stabilization of β-catenin activates classical Wnt signaling, which subsequently induces the expression of numerous genes related to cellular and developmental processes (13). GSK3 is a key mediator in the β-catenin signaling pathway (15). Inhibition of GSK3 by Wnt signaling stabilizes many proteins in a process named WNT-STOP. Wnt signaling is highly active in the G2/M phase of the cell cycle (16). Thus, WNT-STOP mediates the process of cell division by increasing the cell size for division (17).

The use of developmental agents such as Wnt ligands to restore morphogenesis at amputated sites is a suitable method to improve mammalian limb regeneration (18). Previous studies showed gain- and/ or loss-of-function of the Wnt pathway in amphibians during limb development and regeneration is not only necessary (19), but it is also able to promote limb bud growth and elongation (20-22). Although, the importance of key molecules such as WNT and BMP by loss and gain of function experiments is established *in vivo* and neonatal mice models (23), assessing these factors in adult mice is difficult due to the lost ability of limb regeneration1-2 weeks after birth (24). For instance, in the proximal amputation of the mouse digit tips as a model system for limb regeneration in mammals, just wound epidermis occurs, connective tissue repair and the bone as the main elements of digit organ, are not regenerated (25). 

In numerous studies, the Wnt signaling pathway was manipulated genetically or through WNT-agonism and inhibitors such as CHIR99021 and IWP-2 small molecules, and its effect was evaluated on tissue and organ regeneration such as limb or digit (20, 26, 27). CHIR99021 is a chemical compound that acts as an inhibitor of the enzyme Glycogen Synthase Kinase 3 (GSK-3) in Wnt signaling and leads to pharmacological activation of the canonical Wnt-signaling pathway. When CHIR99021 banded to the Wnt receptor named disheveled (Dvl/Dsh), the inhibition of the GSK3 caused the β-catenin destruction complex. This leads to accumulation of free non-phosphorylated β-catenin in the cytosol then to the nucleus and transactivates Wnt-target functions such as cell proliferation and osteogenesis (28). Inhibitor of Wnt Production 2 (IWP-2) (IC_50_ = 27nM) is a Wnt pathway inhibitor that acts by inhibiting disheveled-2 (Dvl2) a Wnt-Fz signaling receptor and inactivating the Porcupine O-acyltransferase (PORCN) which palmitoylates Wnt proteins and inhibits self-renewal and cell proliferation in culture (28). 

Here we use the CHIR99021 chemical to find the Wnt signaling effect on digit tip regeneration of mice *in vitro*. Also, IWP-2 was utilized for confirmation of CHIR99021 effects.

The novel *ex vivo* biological systems have furthered the development and availability of three-dimensional organoid cultures such as 3D cultures, organoids, or organ chips (organs-on-chips) (28). These systems have advanced repeatability, accuracy, and control of the tissue or organ microenvironment, used for other multi-tissue organ experiments such as heart, liver, and kidney (29, 30).

A system that effectively models the complexity of limb development does not exist. However, the whole organ culture system is a powerful model system, an ideal and accessible laboratory system for limb/digit tip *ex vivo* studies (31, 32). In this system, the focus is on one intended factor and the effect of other non-specific factors is eliminated. In addition, tissue organization, differentiation and controlled cellular events occur (33). Although, novel *ex vivo* investigations such as 3D cultures, organoids, or organ chips (organs-on-chips) which have advanced repeatability, accuracy, and control of the tissue or organ microenvironment, are used for other multi-tissue organ experiments such as heart, liver, and kidney (29, 30).

So, in the current study, we focus on the canonical Wnt/β-catenin pathway effect on digit tip regeneration into amputated limb bud organ culture system. 

## Materials and Methods

***Ethical statement***

The Royan Institutional Review Board and Institutional Ethics Committee of Royan Institute (No: EC/93/1028) approved this study. All experimental procedures were conducted under the standard guidelines of the NIH Guide for the Care and Use of Laboratory Animals (34).

***Animals and organ harvest***

The present study is an experimental study in which 6–8-week-old C57BL/6J males and females were used for mating. We used 5 pregnant C57BL/6J females (approximately 25 fetuses) in this study. The animals were housed in stable laboratory situations with a temperature of 22 °C and 50 % humidity, a 12-hr day/night cycle, and free access to food and water. To harvest the cultured hand organ, the 14.5, 16.5, and 18.5-day-old pregnant mice were anesthetized with 80 µl of ketamine and 120 µl of xylazine (35). All fetuses were collected and euthanized with an intraperitoneal injection of 0.08 ml normal saline containing pentobarbital sodium (390 mg/ml), after deep anesthesia with CO_2_ for 35 min (36), then their forelimb wrists were amputated following anesthesia, under aseptic conditions.

***Digit tip amputation and organ culture***

The model of digit tip amputation and 7-day culture was carried out as explained in our previous study (35). Briefly, we amputated about 1–2 mm at the proximal site of digits 2, 3, and 4 of the forelimbs under a microscopic loop to remove any regenerable levels of digit tips. The amputated paws were placed on a 70 µm filter insert mesh and subsequently transferred to 24-well tissue culture plates that contained Dulbecco’s Modified Eagles Medium (DMEM; Sigma-Aldrich) supplemented with 15% fetal bovine serum (FBS; Gibco; Germany), along with 10000 units of penicillin and 10 mg/ml streptomycin (Sigma-Aldrich; Germany) to prevent bacterial contamination of the digit cultures. The growth medium was refreshed twice a week for 7 days. 

***Treatment of digit with CHIR99021 and IWP-2 chemicals***

Samples were randomly divided into three groups to determine the effective dose of CHIR99021 and IWP-2 chemicals and the control group. We selected 10 and 4 different concentrations of CHIR99021 and IWP-2 chemicals based on literature, respectively (37). The samples were then cultured with growth culture medium supplemented with selected CHIR99021, IWP-2 concentration as experimental groups, and DMEM without treatment, as a control group for 7 days of cultivation. To determine the elongation of tissue regrowth or fingertip regeneration, the newly formed clear areas in the fingertips were measured after 7 days. The used concentrations of CHIR99021 and IWP-2 chemicals are represented in Supplementary Table 1.

***Whole mount analysis***

To ensure the positive effect of 25 and 30 μM/ml concentration of CHIR99021 on digit tip regrowth, we compared 3 experimental groups of 25 and 30 μM/ml, and Nrm Reg with Intact as a control group after 7 days. The paws were collected for investigating of growth or shrinkage ratios of the digit tips at 7-day post-amputation (7 dpa). The cultured samples in DMEM were used as a control group (Nrm Reg). To determine the amount of digit tip elongation, digits were fixed overnight at 4 °C in a 10% formalin solution. After washing with 1% potassium hydroxide (KOH) in H2O, the digits were serially incubated in 20%–100% glycerol/1% KOH for 4–16 hr. at 37 ºC. The samples then were assessed with an inverted microscope. Approximately 15 random digits were used for each group. 

Digit tip regeneration was measured by 4 parameters of whole mount (bone elongation) and histological (new tissue formation, cell proliferation, and epidermal closure) analysis. Bone/digit elongation was analyzed using the measurement software of a P-63 Camera (Canon, Japan) (Table 2). Also, cell proliferation and new tissue formation were evaluated using specific gene expressions such as P63, CAN, and Keratin14.

***Histological analysis***

*Hematoxylin and eosin (H* *&* *E), and alcian blue (AB) staining*

After fixation of samples in 10% formalin solution and routine histological process, the 5–6-µm thick sections were prepared and stained with gill’s hematoxylin to stain the nuclei and acidified eosin to counterstain the cytoplasm according to standard procedures (H&E). To detect the cartilage segments, we stained some sections with AB for cartilage. Also, for new tissue regrowth, we measured the length of each distal phalanx by measurement software of DP2-BSW.

***Real-time PCR measurement***

The expression levels of WNT-associated genes included the transcription factor *Tcf1*, a key transcription factor Also, a downstream effector of WNT/*β-catenin* signaling (38) and β-catenin in CHIR treated group, and amputated digit tips (without CHIR treatment) as a control group were evaluated by quantitative-reverse transcription-polymerase chain reaction (qRT-PCR). Briefly, total RNA was extracted from the cells by manual RNA extraction with Trizol (Life Technologies). cDNA was produced by the RevertAid First Strand cDNA Synthesis Kit (Fermentas, USA) according to the manufacturer’s instructions. qRT-PCR reactions were completed in triplicate using SensiMix™ SYBR® (Applied Biosystems Life Technologies, Inc., ref: 4367659) with the ABI StepOnePlus real-time PCR system (Applied Biosystems Life Technologies, Inc.) and then analyzed with StepOne software (Applied Biosystems Life Technologies, version 2.1; USA). The reference gene GAPDH was used to normalize the expression levels of the genes. We used the comparative ΔΔCT method for data analysis. Table 1 lists the primers used in the experiments. 

***Immunohistochemistry (IHC) evaluation***

Immunohistochemistry (IHC) was used to evaluate Wnt signaling pathway factors (TCF1, β-Catenin), the epidermis (Keratin-14), and cell proliferation factor (P63) or cartilage markers (Acn). Briefly, 10% BSA with 2% goat serum was used for 30 min to block any nonspecific antigens, followed by overnight incubation with primary antibodies TCF1 (ab176376, USA), β-catenin (orb48101, UK), P63 (ab110038, USA), Keratin-14 (sc32758, USA) and ACAN at 4 °C. The secondary antibody goat anti-mouse (A0168, Sigma) was used at 1:5000 concentrations for 1 hr. The results were observed by a light microscope (Olympus, Japan).

***Statistical analysis***

Statistical analyses were performed on datasets of at least three independent experiments using an unpaired student’s t-test when comparing two groups. One-way ANOVA with Tukey’s multiple comparison tests was used to compare more than two groups and GraphPad Prism software (GraphPad, San Diego, CA, USA).

**Table 1:** The used concentration of CHIR99021 and IWP-2 chemicals and measurements of regenerated digit tips following treatment of digit tips

**Target gene**	**Primer sequence**	**Accession number**	**Annealing temperature**	**Product size**
**Tcf 1**	F: 5' TACCCTACAAATGCTTCTCCTG 3' R: 5' AAACGTATCCTAGTCCCTCCT 3'	NM_009327.3	60 ⁰C	247 bp
**β-catenin**	F: 5' AGAACACTAATTCATAATCACGCT 3' R: 5'GGCTCAAATAACACCTCTTACTG 3'	NM_001165902.1	60 ⁰C	209 bp
**GAPDH**	F: 5’ GACTTCAACAGCAACTCCCAC 3’ R: 5’ TCCACCACCCTGTTGCTGTA 3’	NM_008084	60 ⁰C	125 bp

**Table 2:** The list of primer sequences relevant to genes of the Wnt signaling pathway

Concentration µm	Chir 1	Chir 3	Chir 6	Chir 10	Chir 15	Chir 20	Chir 25	Chir 30	Chir 35	Chir 40	IWP2 5	IWP2 10	IWP2 15	IWP2 20
New growth length means	24.96	31.87	50.05	60.31	101.65	112.90	186.59	207.23	58.08	48.65	58.38	100.12	70.14	51.17
Standard deviation	+/- 1.08	+/- 4.30	+/- 11.86	+/- 3.24	+/- 28.51	+/- 37.79	+/- 23.36	+/- 41.77	+/- 14.67	+/- 3.25	+/- 7.77	+/- 4.35	+/- 6.57	+/- 7.16

Tcf1: T caell factor 1, GAPDH: glyceraldehyde-3-phosphate dehydrogenase

**Figure 1:**
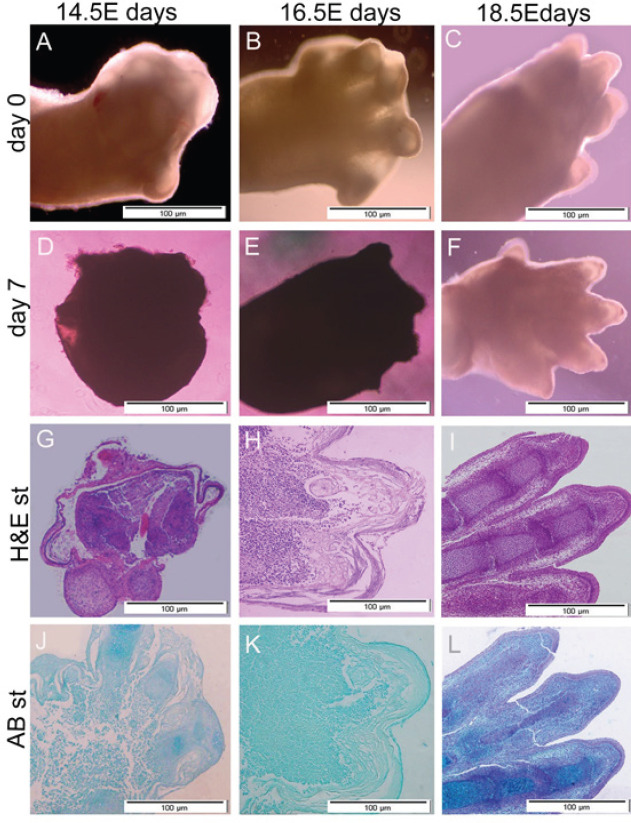
Organ culture of embryo paws of 18.5E day old C57BL/6J mice

A-C) Whole mount hands culture at 14.5E, 16.5E, and 18.5E days at day 0. D-F) Representative images of the hand culture after 7 days, shrinkages of tissues at 14.5E and 16.5 were observed in the hands cultured after 7 days. G-I), H&E staining shows normal and degenerated tissues in 3 stages of hands cultured. J-L) Alcian blue staining shows normal and degenerated skeletal elements during 7 days at 3 stages. . E: Stage of embryo ages 14.5. 16.5 and 18.5 days. Scale Bar: 500 µm.

**Figure 2:**
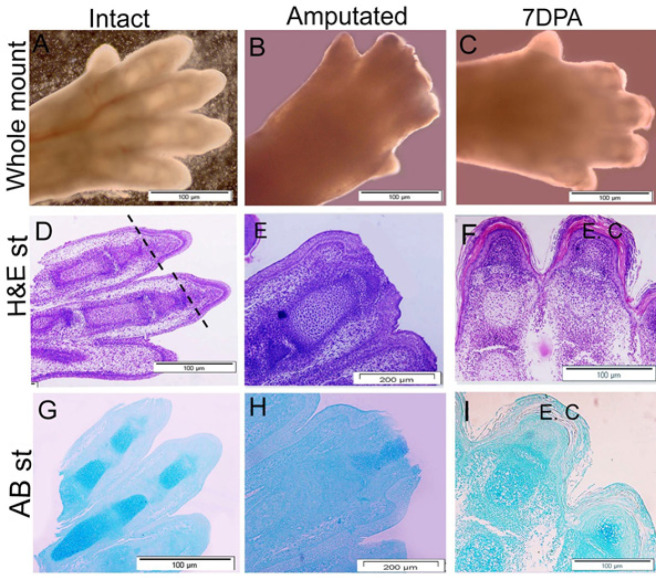
Model of non-regenerative proximally amputated 18.5E mouse digit tip

A) Intact control, embryo hand at 18.5E. B) Embryo hand at 18.5E, immediately after digit amputation. C) Embryo hand at 18.5E, 7 days after digit amputation. D-F) Hematoxylin and eosin (H&E) staining shows the exact amputation site of the digit tip. (E) Epidermal closure after 7 days (F). G-I) Alcian blue staining shows tissues and skeletal elements in Intact (G), immediately amputated (H), and 7-day cultured models (I). E. C: Epidermal closure. The dotted line shows the amputated site

**Figure 3:**
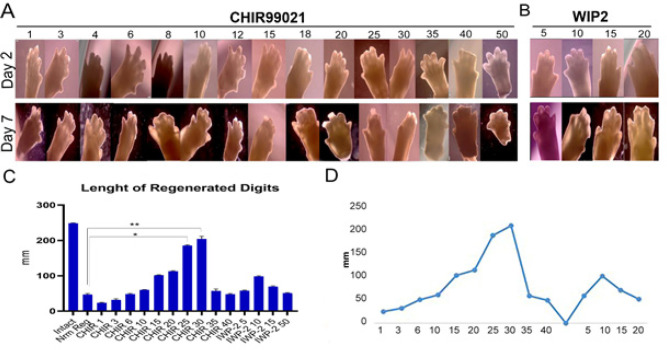
Evaluation of complete digit tip in different concentrations of CHIR99021 and IWP2 after 2 and 7 days

A) Image representatives of whole mount digit tips at various concentrations of CHIR99021 and B) IWP2 on days 2 and 7 after amputation. C) Measurement of bone elongation in various concentrations of CHIR99021 and IWP2 after 7 days. D) Histogram shows the gradual process of bone length measurements for all experimental groups. Data are presented as means ± SD. The comparisons were performed using one-way analysis of variance (ANOVA). Nrm Reg: Normal Regeneration group. (n=3), ** *P*<0.01, * *P*<0.1.

**Figure 4:**
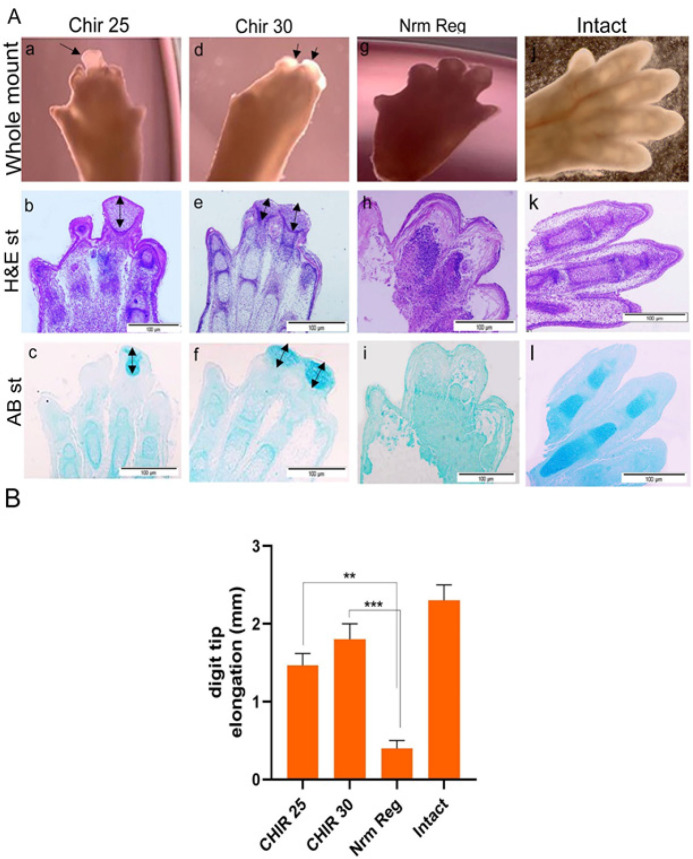
Evaluation of CHIR99021 effect on the regeneration of amputated digit tip at different concentrations

A) Whole-mount and histological analysis of concentrations 25 and 30 l/ml of CHIR99021 on regeneration of the amputated digits, images illustrate new tissues and skeletal elements such as cartilage in H&E and Alcian blue staining; CHIR 25 (a-c), CHIR 30 (d-f), Nrm Reg (g-i), and Intact (j-l). B) Histogram shows a significant increase of digit elongation in CHIR 25 and CHIR 30 compared to the Nrm Reg groups. Data are presented as the mean ± standard deviation (n=10). The comparisons were performed using One-way analysis of variance (ANOVA). ***P<0.001. Arrows show the site of new tissue formed. Nrm Reg: Normal regeneration in an amputated group without treatment.

ns: Non-significant

**Figure 5:**
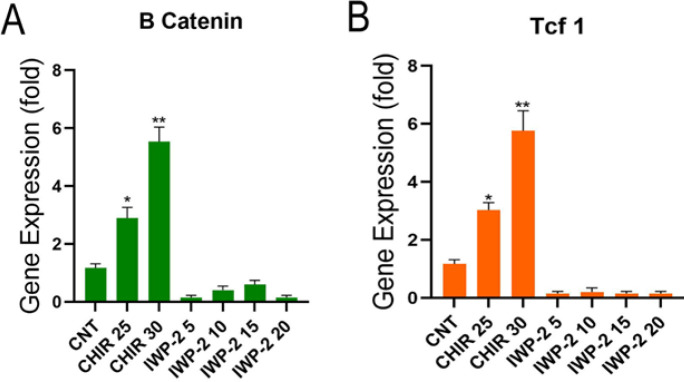
qRT-PCR analysis of WNT signaling mediator gene expression A) Histogram shows a significant increase in B catenin gene expression in 25 and 30 µl/ml concentrations of CHIR99021. B) Tcf1 expression was also observed in 25 and 30 µl/ml concentrations of CHIR99021. Data are presented as the mean ± standard deviation (n=5). Comparisons were performed using two-way analysis of variance (ANOVA)

Cnt group: Amputated digits without CHIR treatment. *P<0.1

**Figure 6:**
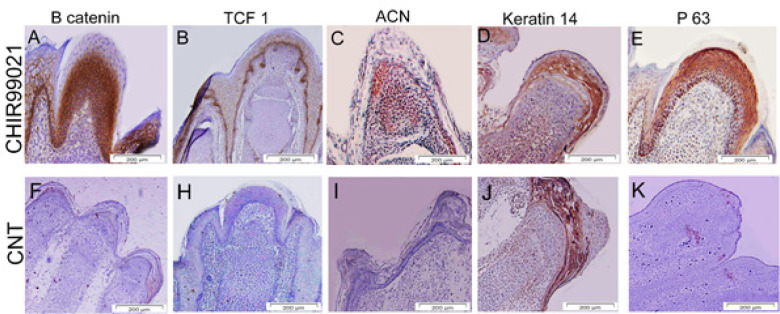
Immunohistochemistry analysis of mice digit tips regeneration following treatment with CHIR99021

IHC staining shows that B-catenin (A) and TCF1 (B) as two key gene mediators of the WNT signaling pathway expressed in the regenerated digits, CAN (C) gene as a cartilage-related genes expressed in new tissue formed, expression of two genes of the Keratin 14 (D), and main factor of dermis and nail formation and P63 (E) a proliferation-related gene also observed in new tissues formed in the CHR99021 group. These gene expressions were also analyzed in the non-CHIR99021-treated group as a CNT group (F-K), scale bar for all figures: 100 µm, CNT: Control

## Results

***Proximal phalanx amputations, culture, and morphological analysis***

As results show in Figure 1 D-E, the paws of E14.5 and E 16.5 were shrunk and compacted after 7 days. The results of H&E staining confirmed that tissues of E14.5 and E 16.5 paws lost the normal structure, and disorders were observed in cartilage, dermis, and epidermis tissues (Figure 1G, H, J, K). While the cells of E18.5 paws were alive after 7 days (Figure 1F) and the clear structures such as cartilage, dermis, and epidermis tissues were completely normal (Figure 1I, L). As illustrated in Figure 2A-C, cultured paws containing intact digit (A), immediately after amputated digit tip (B) and regrowth digit 7 DPA (C) were alive and normal. The H&E staining shows a clear structure such as cartilage mass, joint and epidermis, and dermis tissues in intact samples (D), the amputate line showed the removed tissues consist of skeletal elements, epidermis and dermis tissues (D), and the new regrowth tissues after 7 days consisting of cartilage mass and epidermis and dermis layers (F). Also, the cartilage mass formation was confirmed by AB staining (G-I). 

***Digit tip regeneration using CHR99021***

As demonstrated in Figure 3 A, at concentrations less than 20 µM/ml of CHR99021 (4, 6, 8, 10, and 15 µM/ml), no new digit tip regrowth was observed. However, tissue deformation or regression tissue did not exist in the digits cultured. In the 25 and 30 µM/ml concentrations of CHR99021, tissue formation and cell proliferation were observed. Conversely, in the 40 and 50 µM/ml concentrations of CHR99021, the regression or compaction of tissue and death cells appeared. The result of IWP-2 treatment shows no new tissue regrowth occurred at 15 and 20 µm/ ml concentration of IWP-2. To determine the elongation of tissue regrowth or fingertip regeneration, the newly formed clear areas in the fingertips were measured after 7 days (the results are shown in Table 1). According to Figure 3 B, significant new tissue formation was observed at 25 and 30 µM/ml concentration of CHR99021. In addition, the regrowth progress of tissue and cell proliferation was shown in Figure 3 C, and the inhibition of tissue formation or tissue regression was confirmed at 15 and 20 µm/ ml concentrations of IWP-2. So, the 25 and 30 μM/ml concentrations of CHIR99021 were selected for further studies.

***Whole mount, histological, and digit tip elongation measurements***

To ensure In order to validate the positive effect of 25 and 30 μM/ ml concentration of CHIR99021 on digit tip regrowth, we compared 3 experimental groups of 25 μM/ ml, 30 μM/ ml, and Nrm Reg with Intact serving as athe control group. In the whole mount analysis, arrows show the new tissue formation in the 25 μM/ ml, and 30 μM/ ml groupgroups (Figure 4. a, d), and new tissue regrowth was not observed in the Nrm Reg group in compared to the intact group (Figure 4. g, j). In H&E assessments, the normal tissues of digits such as the epidermis, dermis, joints, and connective tissue were alive, and dead cells or shrinkage regions did not appear in the intact group (Figure 3. k) after 7 DPA. In the Nrm Reg group, just an epidermal wound occurred and no cell proliferation or new tissues such as cartilage mass were observed (Figure 4. h). The AB staining confirmed the new regrowth areas that colored blue were cartilage-like-tissue (Figure 4 c, f). In addition, an assessment of digit elongation showed in Figure 4 B, that amputated digits in concentrations of 25 μM/ ml, and 30 μM/ ml CHIR99021 groups significantly were elongated in comparison to Nrm Reg. As well as in both the 25 and 30 μM/ml groupgroups, the arrows detected the new tissue formed 7 DPA (Figure 7. b, e). 

***WNT-related gene analysis***

qRT-PCR analysis of WNT-related genes showed significant differences in the expression levels of tcf1 and ß-catenin in regenerated fingertips after 7 days. In detail, tcf1 and ß-catenin genes show a higher expression in doses of 25 and 30 μM/ml concentrations of CHIR99021 compared to the control group (Figure 5A, B). The ability of inhibition of tcf1 and ß-catenin gene expression to undergo IWP-2 was assessed by qRT-PCR analysis. The results of IWP-2 analysis indicated a rare expression of tcf1 and ß-catenin genes compared to the control group (Figure 5A, B) after 7 days. There was no considerable difference in the expression level among tcf1 and ß-catenin genes in all concentrations.

***β*** ***catenin, TCF 1, Keratin 14, ACN, and p63 protein expression analysis***

As illustrated in Figure 6, β catenin and TCF 1 up-regulated in newly formed epidermal and mesodermal tissue, which are the main markers of Wnt signaling in regrowth tissue in limb regeneration (A, B). We observed the expression of ACN, as the main protein of cartilage tissue factor, in newly tissues formed (C). Also, Keratin-14 as the main skin marker was expressed in this group (D). Our data demonstrate that in 30 Mμ/ml concentrations of the CHR99021 group, the P63 gene’s high expression confirmed the wild range of cell proliferation in samples that received CHR99021 (E). However, β catenin, TCF 1, ACN, and p63 protein expressions were not observed in Nrm Reg as a control group (Figure 6F-I, K). Nonetheless, the Keratin 14 expression was observed in the control group due to the epidermis closure that occurred in the control group (J).

## Discussion

In the current study, we designed an organ system consisting of the forelimbs of 18.5E old C57BL/6J fetal mice then used two chemicals, CHIR99021 and IWP-2, to active or inhibit the Wnt signaling pathway to investigate the efficiency of this signaling pathway for tissue regeneration. Our findings provided evidence for the functional roles of CHIR99021 in the regenerative process of amputated digit tip of fetuses obtained from mice. Also, IWP-2 inhibits cell proliferation and also digit tip regeneration. 

In response to limb or digit tip amputation in mammalians, regeneration occurs just in skin tissue and not in the musculoskeletal tissues of mice due to scar formation (39). Wnt signaling is a key factor during limb development that is activated in the wound healing process and controls the inflammation responses and programmed cell death, proliferation, and cell differentiation (40). The importance of the Wnt signaling pathway during limb development and tissue homeostasis causes Wnt pathway to be one of the main candidates for translational applications during limb/appendage regeneration (41-43). Previous studies have shown inhibition of Wnt signaling causes amphibian limb regeneration failure (20, 22, 44). For instance, Singh *et al*. showed that hedgehog (HH) signaling is correlated with Wnt signaling as key factors for blastema cell formation, proliferation, and cell remodeling during newt limb regeneration (45). They investigated that inactivation of the HH pathway leads to blastema formation failure, nevertheless, activation of Wnt signaling enhanced proliferative signals and rescued the inhibition of the HH pathway largely (26). 

Wnt-dependent osteogenesis and cell proliferation are two main functions that were regulated by enhancing Wnt signaling (45, 46). Our histological result demonstrated that the new tissue formed in the CHIR99021 group following up-regulation of Wnt signaling, not in the control or IWP-2 groups. These results confirmed the importance of Wnt signaling necessary in digit tip regeneration.

Another study showed that treating digit amputation wounds with BMP9 in neonatal mice stimulates regeneration of a synovial joint that forms an articulation with the stump bone (46). Takeo *et al*. demonstrated Wnt signaling activation is required for nail regeneration in amputated digit tips of adult mice. They showed that the proximal nail matrix homed the nail stem cells (NSCs) that promote mesenchymal blastema growth and nail formation. So, Proximal amputations cause the failure of the nail/digit regeneration due to lost NSCs. Then, they rescued nail repair by treating K14-CreER-β-catenin flex3 mice with TAM. Following β-catenin stabilization in the NSC region, the nail regenerated (47). 

Here, we try to evaluate the effects of Wnt signaling using CHIR99021 on appendage regeneration in the organ culture system, we demonstrated that this pathway is not only necessary for digit tip regeneration but is also able to promote regeneration in neonatal mouse digit tip. More importantly, we showed that digit tip regeneration occurred in a controlled 3-dimensional organ culture system. It should be noted that in this organ culture system, conditions of culture are more controllable than *in vivo* models due to the limitation of culture media and activators or inhibitors and lack of cell interactions (48, 49). 

 On the other hand, the organ culture system is a traditional but powerful system for exploring related limb regeneration (50-53). In this method, the manipulation of signaling pathways, examination of gene expression, and longitudinal lineage tracking are available (54). Recently, researchers reported a protocol related to embryonic limb bud organ culture due to necessitating the use of limb cultures for investigation of musculoskeletal development and regeneration, as well as its challenges of limb organ culture (55). The current study used an organ-cultured system for *ex vivo* amputated analysis. In this system, the viability of cultured organs during time culture is a very important issue. For instance, the size of the cultured organ is one of the main factors that control the viability of cultured organs, the release of nutrients and oxygen is related to organ size (56). So, we examined 14.5, 16.5, and 18.5 E day fetuses to find the best size of paw organ culture. Also, the amputated paw of the 18.5 E day embryo was alive after 7 days, wound closure occurred, and no dead cells were observed. Therefore, we selected the 18.5 E day embryo for our experiments (Figure 1F). Our results show the digits of 14.5 and 16.5 E day fetuses were too small to be manipulated and also their connective tissue was very loose. So, the tissues showed shrinkages and dead cells appeared after 7 days (n=3) (Figure 1D, E). Also, previous studies demonstrated that maintenance of primary embryos in culture challenges tissues and they became shrunk and compressed in *in vitro* conditions after several days. They showed these embryos are more sensitive in comparison with older embryos and died in the first days after culture (57, 58). Although we do not know the underlying mechanism of the non-establishment of cultured paws and embryos before the stage 18 embryo *in vitro*, the results of cultured paw in the current study confirmed their results.

Researchers showed GSK-3β, an Inhibitor of CHIR99021 promotes osteogenesis by activating Canonical Wnt signaling in bone marrow stromal cell ST2 and promotes osteoblast differentiation and mineralization (59). So, we used various concentrations of CHR99021 to find the effective dose for 3D regeneration of amputated digits. As results showed in Figure 3, the 25 and 30 µM/ml concentrations of CHR99021 are effective doses for new bone or cartilage tissue formation, as well as cell proliferation and digit elongation. The 40 and 50 µM/ml concentrations of CHR99021 showed a negative or toxic effect on organs and caused the regression or compaction of tissue and cell death. The regrowth progress of tissue and bone elongation was shown in Figure 3 C, and the inhibition of tissue formation or tissue regression was confirmed at 15 and 20 µm/ml concentrations of IWP-2. So, the 25 and 30 μM/ml concentrations of CHIR99021 were selected for further studies. However, new tissue regrowth, digit elongation, and cell proliferation did not show a significant difference in 25 and 30 μM/ml concentrations of CHIR99021 (Figure 4). To ensure the selection of concentrations of CHIR99021, we decided that two 25 and 30 μM/ml concentrations were used for WNT signaling mediator gene expression. qRT-PCR showed that BC and Tcf1 genes significantly expressed in 30 μM/ml concentration of CHIR99021 more than 25 μM/ml concentration of CHIR99021. We did not identify the cause of this result, so this needs more experiments. Lu *et al*. showed that osteocytes treated with CHR99021 formed an osteogenic microenvironment called (COOME). They then designed a bio-instructive 3D system by combining COOME with osteoblast cells (ST2) to mimic the *in vivo* environment of bone. This system increased the survival and proliferation rate of ST2 cells to 92% after 7 days, promoting differentiation and mineralization. It also promotes HUVEC migration and tube formation, which can be related to the high expression of the VEGF gene (60). Since angiogenesis is considered an essential process of cell growth in 3D culture systems, its investigation is of great importance. However, in the organ system, we did not investigate angiogenesis due to a small system. 

On the other hand, the Wnt inhibitor, IWP-2, inhibits Smad1/5 and eventually the Wnt signaling pathway. IPW2 inhibits various cellular actions such as cell proliferation, differentiation, and EMT (61). We then confirmed that the Wnt inhibitor, IWP-2, was effective in our phalange culture system. The result of IWP-2 treatment shows no new tissue regrowth occurred at 15 and 20 µm/ ml concentrations of IWP-2 treated groups. Finally, we performed IHC staining for the presence and location of specific proteins of β catenin, TCF 1, Keratin 14, ACN, and p63 in the tissue sections. We selected the concentration of the 30 μM/ml CHIR99021 group as the best of the experimental groups and Nrm Reg as the control group. The immunohistochemistry outcomes showed that activation of beta-catenin signaling in amputated digits in our organ culture system causes new tissue formation or increases the cell proliferation that showed by p63 gene expression, ACN, a main cartilage-related gene, as well as, keratin-14 gene as a key gene of nail formation.

## Conclusion

This study emphasizes the use of an organ culture system to establish a limb organ culture, as well as a Wnt signaling pathway for limb regeneration. Organ cultures using animal organs reproduce mammalian conditions and are a useful tool to test gain and loss responses to drugs or therapeutic agents. This model showed amputated limbs at 18.5 E are alive. In addition to the scientific aims limb organ culture showed that the Wnt signaling pathway is a main factor of digit tip regeneration in mice. So, our studies may provide valuable insights toward a better understanding of adult tissue, such as limb/digit, regeneration. 

## Name of the Institutional Approval Committe ot Unit

Research Ethics Committee of Royan Institute (Approval number: EC/93/1028).
